# Microbial and Metabolic Correlates of Endometrial Dysfunction in Polycystic Ovary Syndrome: A Translational Study

**DOI:** 10.1111/1471-0528.70266

**Published:** 2026-05-19

**Authors:** Mengyu Jing, Xi Chen, Meiyan Jiang, Huiya Fang, Xiaoming Zhu, Xuejing Jin, Yonghui Jiao, Ningning Hou, Weiwei Gong, Aixia Liu

**Affiliations:** ^1^ Department of Reproductive Endocrinology, Women's Hospital, School of Medicine Zhejiang University Zhejiang Hangzhou People's Republic of China; ^2^ Key Laboratory of Reproductive Genetics (Ministry of Education) Zhejiang University Zhejiang Hangzhou People's Republic of China; ^3^ Department of Ultrasound, Women's Hospital, School of Medicine Zhejiang University Zhejiang Hangzhou People's Republic of China; ^4^ Assisted Reproduction Unit, Department of Obstetrics and Gynecology, Sir Run Run Shaw Hospital, School of Medicine Zhejiang University Hangzhou People's Republic of China; ^5^ Department of Reproductive Medicine Center Hangzhou Women's Hospital Zhejiang Hangzhou People's Republic of China; ^6^ Department of Reproduction People's Hospital of Xinjiang Uygur Autonomous Region Urumqi People's Republic of China; ^7^ Department of NCDs Control and Prevention Zhejiang Provincial Center for Disease Control and Prevention Hangzhou People's Republic of China; ^8^ Institute of Medical Genetics and Development Zhejiang University Zhejiang Hangzhou People's Republic of China

**Keywords:** endometrial senescence, gut microbiota, isoleucine, miscarriage, polycystic ovary syndrome

## Abstract

**Objective:**

Women with polycystic ovary syndrome (PCOS) exhibit a substantially increased risk of miscarriage, yet the underlying mechanisms remain inadequately understood. This study aimed to investigate whether specific gut microbial dysbiosis and metabolic disturbance are associated with and may potentially contribute to endometrial dysfunction and adverse pregnancy outcomes in women with PCOS.

**Design:**

Prospective cohort study integrated with mechanistic experiments.

**Setting:**

Women's Hospital, School of Medicine, Zhejiang University, China (2022–2024).

**Population:**

A total of 110 women with PCOS and 110 age‐ and body mass index‐matched controls were enrolled.

**Methods:**

We performed 16S rRNA and metagenomic sequencing of gut microbiota, with untargeted and targeted serum metabolomics. Functional validation was conducted using primary human endometrial stromal cells and a PCOS rat model intervened with *Parabacteroides merdae* ( *P. merdae* ) supplementation or faecal microbiota transplantation.

**Main Outcome Measures:**

Gut microbiota composition, serum metabolites, endometrial senescence markers, and pregnancy outcomes.

**Results:**

Women with PCOS exhibited significantly higher miscarriage rates than controls, accompanied by a marked depletion of *P. merdae* abundance and elevated serum levels of branched‐chain amino acids, particularly isoleucine. Exogenous isoleucine induced cellular senescence in human endometrial stromal cells in a dose‐dependent manner. Restoration of *P. merdae* levels in the PCOS rat model resulted in decreased serum isoleucine levels, amelioration of the senescent endometrial phenotype, and reduction in the fetal resorption rate.

**Conclusions:**

These findings suggest that *P. merdae* depletion and the concurrent accumulation of isoleucine may be associated with endometrial senescence and elevated risk of miscarriage, suggesting the possible involvement of a gut microbiota–metabolite pathway in PCOS‐related reproductive dysfunction. These results also provide a mechanistic basis for future translational investigations.

## 1 Introduction

Polycystic ovary syndrome (PCOS) affects approximately 6%–10% of women of reproductive age and is one of the leading causes of infertility [1]. Current international guidelines attribute PCOS‐related infertility primarily to oligo‐ or anovulation, directing reproductive therapeutic strategies predominantly towards restoring ovulatory function [2, 3]. While a comprehensive review of the literature reveals insufficient evidence to implicate intrinsic oocyte defects as the principal factor underlying the reduced fertility observed even in ovulatory PCOS women [4]. This paradox underscores the potential contribution of other mechanisms, particularly given compelling clinical evidence indicating significantly higher rates of adverse pregnancy outcomes, notably an increased risk of miscarriage, among women with PCOS experiencing spontaneous ovulation [5]. However, the precise molecular and cellular mechanisms by which PCOS predisposes to miscarriage remain largely elusive.

PCOS is recognized as a multifactorial disorder influenced by metabolic, endocrine, and genetic factors [6]. PCOS patients exhibit characteristic gut microbial dysbiosis [7, 8], closely associated with insulin resistance and hyperandrogenism [9, 10, 11, 12]. Gut‐derived metabolites, particularly branched‐chain amino acids (BCAAs) and secondary bile acids, serve as critical signalling molecules mediating microbiome‐host crosstalk [13, 14, 15]. Recent studies have revealed direct links between gut microbiota and reproductive outcomes: specific gut‐derived amino acids affect pregnancy maintenance in murine models [16], while disrupted microbiota‐metabolite interactions contribute to endometrial dysfunction in women with miscarriage [17, 18]. Despite these advances, the specific contribution of microbial dysbiosis and associated metabolic shifts to the elevated miscarriage risk in PCOS patients represents a significant and unexplored research frontier.

Accumulating evidence points to endometrial dysfunction as one of the key contributing methods to PCOS‐associated pregnancy complications [19]. The PCOS endometrium exhibits multiple molecular abnormalities, including aberrant hormone receptor expression and altered cellular composition [20]. PCOS is characterized by chronic low‐grade inflammation [21], recognized as a potent inducer of cellular senescence [22]. Recent single‐cell transcriptomic profiling revealed that the PCOS endometrium contains fewer endometrial stromal cells (ESCs) [23]. Moreover, premature or excessive accumulation of senescent ESCs through senescence‐associated secretory phenotype (SASP) factors such as IL‐1β, IL‐6, and IL‐8 disrupts decidual function [24, 25]. However, the upstream triggers that drive endometrial dysfunction or senescence in women with PCOS remain unidentified.

In this study, we aimed to investigate the role of gut microbiota and metabolic disturbances in PCOS‐related reproductive failure. We combined multi‐omics profiling with functional assays to elucidate the underlying mechanisms. We hypothesised that elucidating these mechanisms could contribute to our understanding of PCOS reproductive pathology and potentially inform early metabolic intervention strategies.

## 2 Methods

### 2.1 Study Design and Participants

This prospective cohort study was conducted at the Women's Hospital, School of Medicine, Zhejiang University, Hangzhou, China, with approval from the institutional ethics committee (IRB‐20220168‐R). All participants provided written informed consent.

We enrolled 220 treatment‐naive women under the age of 35 years at their first clinical visit: 110 women diagnosed with PCOS and 110 age‐ and body mass index (BMI)‐matched controls. PCOS was diagnosed according to the 2003 Rotterdam criteria [26, 27], and the distribution of PCOS phenotypes is displayed in Figure S1. Controls were women with infertility due to tubal factors. All participants (PCOS and control groups) received IVF/ICSI treatment under either a GnRH antagonist or agonist protocol, minimizing potential confounding due to differences in treatment regimens. Exclusion criteria are (i) endocrine disorders, (ii) current use of medications affecting gut microbiota (antibiotics, probiotics, or prebiotics within 4 weeks), metformin, or steroidal hormones, (iii) active pelvic inflammation or severe hydrosalpinx. (iv) inflammatory bowel disease or chronic gastrointestinal disorders.

The median follow‐up period for pregnancy outcomes was 16.6 months. Clinical pregnancy was confirmed by transvaginal ultrasound. Following the Chinese clinical practice guideline [28], miscarriage was defined as spontaneous pregnancy loss before 28 weeks of gestation, confirmed by ultrasound examination. Participant clinical characteristics are summarized in Tables S1–S5, with the overall study design and sample allocation illustrated in Figure 1A. Detailed laboratory protocols, bioinformatics pipelines, and additional analytical methods are provided in the Supporting Information.

**FIGURE 1:**
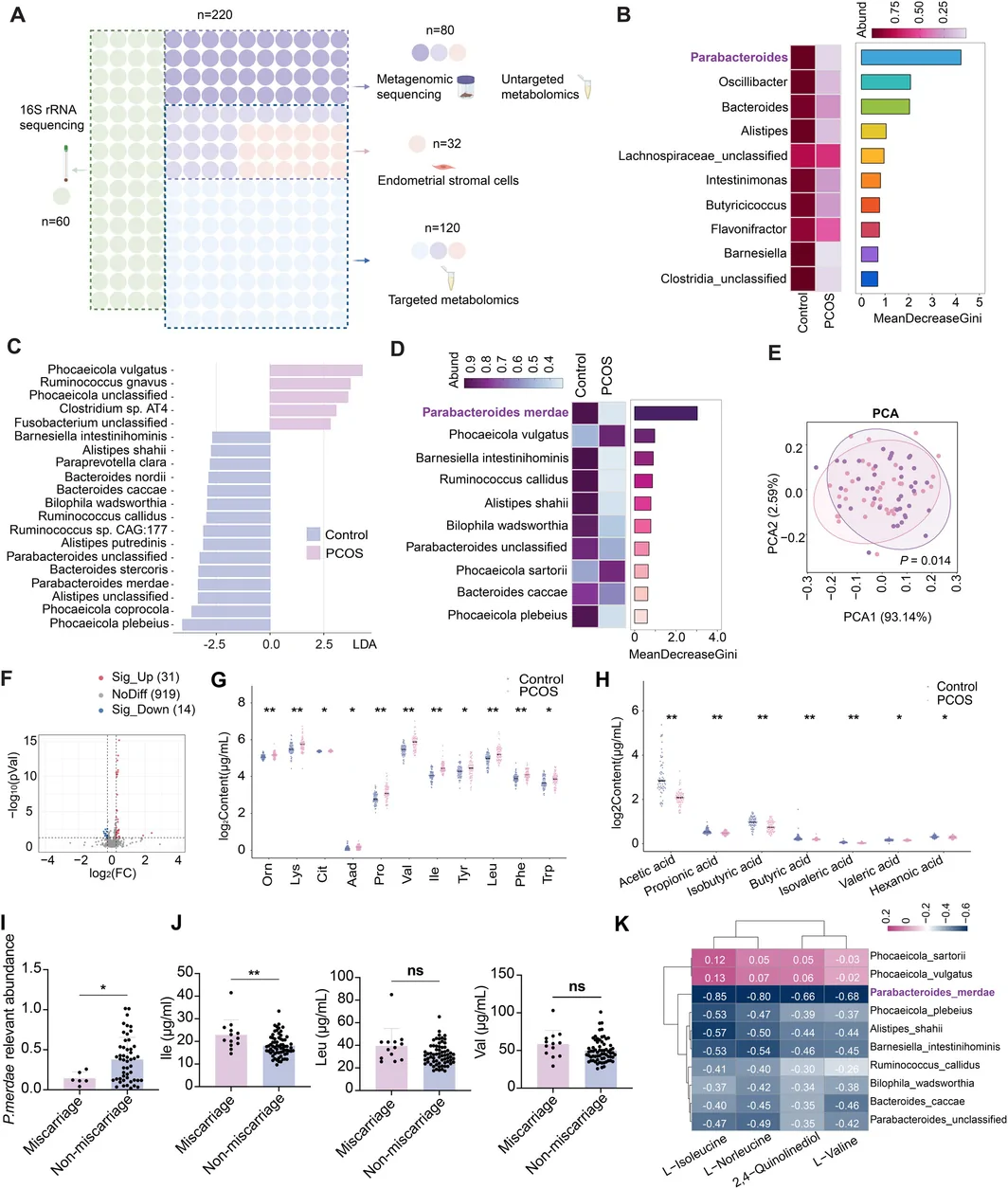
Study design and integrated multi‐omics profiling of gut microbiome and serum metabolites in PCOS. (A) Study design and participant flow chart. (B) Feature importance plot from the random forest model showing mean decrease in Gini index. (C) LDA scores for differentially abundant species (LDA > 2). (D) Random forest feature importance of bacterial species for group classification. (E) PCA score plots. (F) Volcano plots showing differential metabolites between PCOS and control group. (G,H) Differential amino acids and SCFAs in PCOS serum identified through targeted analysis. (I) *P. merdae* abundance in miscarriage and non‐miscarriage groups (PCOS and control groups combined). (J) Serum BCAA concentrations between miscarriage and non‐miscarriage cases (PCOS and control groups combined). (K) Heatmap showing correlations between serum metabolites and bacterial strains across all participants. ns *p ≥ 0.05*, *p < 0.05, **p < 0.01. Figure 1A was created by M.J. using BioRender.com (https://BioRender.com/w9lo5dz).

## 3 Results

### 3.1 Gut Microbiota Profiles in PCOS Are Associated With Hormonal Disturbances

We employed a two‐pronged sequencing approach, consisting of 16S rRNA gene sequencing followed by metagenomic shotgun sequencing, to compare the gut microbial composition between PCOS patients and control women. Significant differences in α‐ and β‐diversity of gut microbiota were observed between PCOS patients and controls using 16S rRNA gene sequencing of faecal samples collected from both groups (Figure S2A–G). *Parabacteroides* relative abundance was significantly reduced and showed the highest discriminatory power at the genus level (Figure 1B). And it was negatively correlated with PCOS‐related indicators (e.g., testosterone [T], luteinizing hormone [LH], or anti‐Müllerian hormone [AMH], Figure S3). Further metagenomic analysis revealed *Bacteroidota*‐dominated microbial ecosystems in both groups, with relative abundances of 56.04% in PCOS and 53.69% in controls, and confirmed significant differences in α‐ and β‐diversity (Figure S4A–E). Importantly, *Parabacteroides* consistently emerged as a significant discriminator, featuring prominently in the metagenomic PLS‐DA model (Figure S4F). LEfSe analysis identified 72 significantly differentially abundant bacterial species (Figure 1C), functionally enriched in pathways including amino acid biosynthesis, ABC transporters, and PI3K‐Akt signalling (Table S6). Among these, *Parabacteroides merdae* ( *P. merdae* ) was markedly downregulated in PCOS and ranked as the most significant species (Figure 1D). The abundance of *P. merdae* was negatively correlated with key PCOS‐related hormones, including T, LH, and the LH/follicle‐stimulating hormone (FSH) ratio (Figure S5).

### 3.2 Serum Metabolomics Reveals BCAA Metabolism Disturbances in PCOS

LC–MS metabolomic profiling of serum revealed distinct metabolic fingerprints differentiating patients with PCOS from controls (Figures 1E and S6A). We identified 45 significantly differentially abundant metabolites, predominantly lipids and organic acids (FC > 1.2 or < 0.83, VIP ≥ 1.0, *p* < 0.05; Figure 1F). Using a combined LASSO and random forest approach, four metabolites were identified as markers: 2,4‐quinolinediol, L‐valine (L‐Val), L‐Ile, and L‐norleucine (Figure S6B,C). These discriminative metabolites were positively associated with AMH, LH, T, and the LH/FSH ratio (Figure S6D).

Complementary targeted metabolomic analysis of amino acids and SCFAs confirmed dysregulation of BCAA metabolism in PCOS serum. Eleven amino acids, including the BCAAs (Ile, leucine [Leu], and Val), were significantly increased (Figure 1G), while five straight‐chain SCFAs (acetic, propionic, butyric, valeric, and hexanoic acids) and two branched‐chain SCFAs (isobutyric and isovaleric acids) were significantly reduced in the PCOS group (Figure 1H). Notably, Ile, Val, and Leu levels correlated positively with T, AMH, LH, and the LH/FSH ratio, whereas acetic acid, isobutyric acid, and isovaleric acid correlated negatively with these indicators (Figure S6E). Furthermore, Ile, representative of the elevated BCAAs, demonstrated negative correlations with seven differentially abundant SCFAs (Figure S6F), most consistently with isobutyric acid (*r* = −0.72) and isovaleric acid (*r* = −0.68).

The relationship between these metabolic alterations and pregnancy outcomes suggested their clinical significance. The median follow‐up duration was 16.6 months (IQR, 12.3–19.8 months). Among all participants who achieved clinical pregnancy (PCOS *n* = 76, control *n* = 77), treatment modalities were comparable between groups, with IVF‐FET being the most common approach (PCOS: 44/76 [57.9%] vs. control: 37/77 [48.1%], *p* = 0.223), and no statistically significant differences were observed across all treatment types (Table S7). The clinical miscarriage rate was significantly higher in the PCOS group compared with controls (20/76 [26.3%] vs. 8/77 [10.4%], *p* = 0.011), while biochemical pregnancy rates (8/76 [10.5%] vs. 2/77 [2.6%], *p* = 0.056) and live birth rates (56/110 [50.9%] vs. 69/109 [63.3%], *p* = 0.064) were also higher in PCOS participants but did not yet reach statistical significance. When analysing pregnancy outcomes of all participants (PCOS and control groups combined), significantly lower *P. merdae* abundance (Figure 1I) and higher serum Ile levels (Figure 1J) were found in subjects who experienced miscarriage compared with those who did not. Similarly, Leu and Val also tended to be higher in subjects with miscarriage than those without, although these differences did not reach statistical significance. These findings were consistent with the negative correlations observed between *P. merdae* abundance and serum levels of L‐Ile, L‐Val, L‐Norleucine, and 2,4‐Quinolinediol (Figure 1K). Receiver operating characteristic (ROC) curve analysis was then performed to evaluate the predictive performance of serum Ile and *P. merdae* abundance for miscarriage. The AUC for Ile was 0.735 (95% CI: 0.594–0.876) and the AUC for *P. merdae* was 0.767 (95% CI: 0.624–0.909), indicating moderate level of diagnostic accuracy (Figure S7). We further compared *P. merdae* abundance and serum Ile levels across Rotterdam phenotypes, focusing on phenotypes A (35.50%) and D (57.30%) as the predominant groups in our cohort. Neither *P. merdae* abundance (*p* > 0.05) nor serum Ile levels differed significantly between the two phenotypes (*p* > 0.05) (Figure S8).

### 3.3 Accumulated Senescence and Functional Impairment in ESCs of PCOS

Transcriptomic sequencing analysis of endometrial tissues obtained during hysteroscopy revealed extensive gene expression changes in the PCOS group (*n* = 5 per group), with 2773 genes significantly upregulated and 4480 downregulated compared with controls (Figure S9A,B). Senescence‐related genes *p53* and *CDKN2A*, along with the amino acid transporter *SLC7A5*, were upregulated in PCOS endometrium. KEGG pathway enrichment analysis indicated that these genes were predominantly enriched in pathways related to cellular senescence, apoptosis, and Val, Leu, and Ile biosynthesis (Figure S9C).

Histological examination showed no overt lesions (Figure S10A). Immunohistochemical analysis revealed prominent cellular senescence in the PCOS endometrium, with significantly higher proportions of cells positive for senescence markers *p21*, *p16* ^*INK4a*^, and SASP factors IL‐6 and IL‐1β (Figure 2A). ESCs isolated from these tissues (identified by vimentin^+^/cytokeratin^−^ immunofluorescence; Figure S10B) from patients with PCOS exhibited enhanced senescence‐associated β‐galactosidase (SA‐β‐Gal) activity, increased SASP factor secretion (IL‐1β, IL‐6, IL‐8), elevated cellular senescence markers, and upregulated DNA damage marker γ‐H2AX (Figure 2B–E). Consistent with this senescent phenotype, PCOS ESCs displayed impaired proliferation (Figure 2F). Ile was significantly elevated in the endometrium of patients with PCOS compared with controls (Figure 2G), and similar elevations were observed in serum (Figure 2H). Furthermore, *P. merdae* was downregulated in the faecal microbiota of these patients (Figure 2I). Significant negative correlations were observed between faecal *P. merdae* relative abundance and endometrial levels of SASP factors (IL‐1β, IL‐6, IL‐8) in ESCs, while serum BCAAs correlated positively with these SASP factors (Figure S11).

**FIGURE 2:**
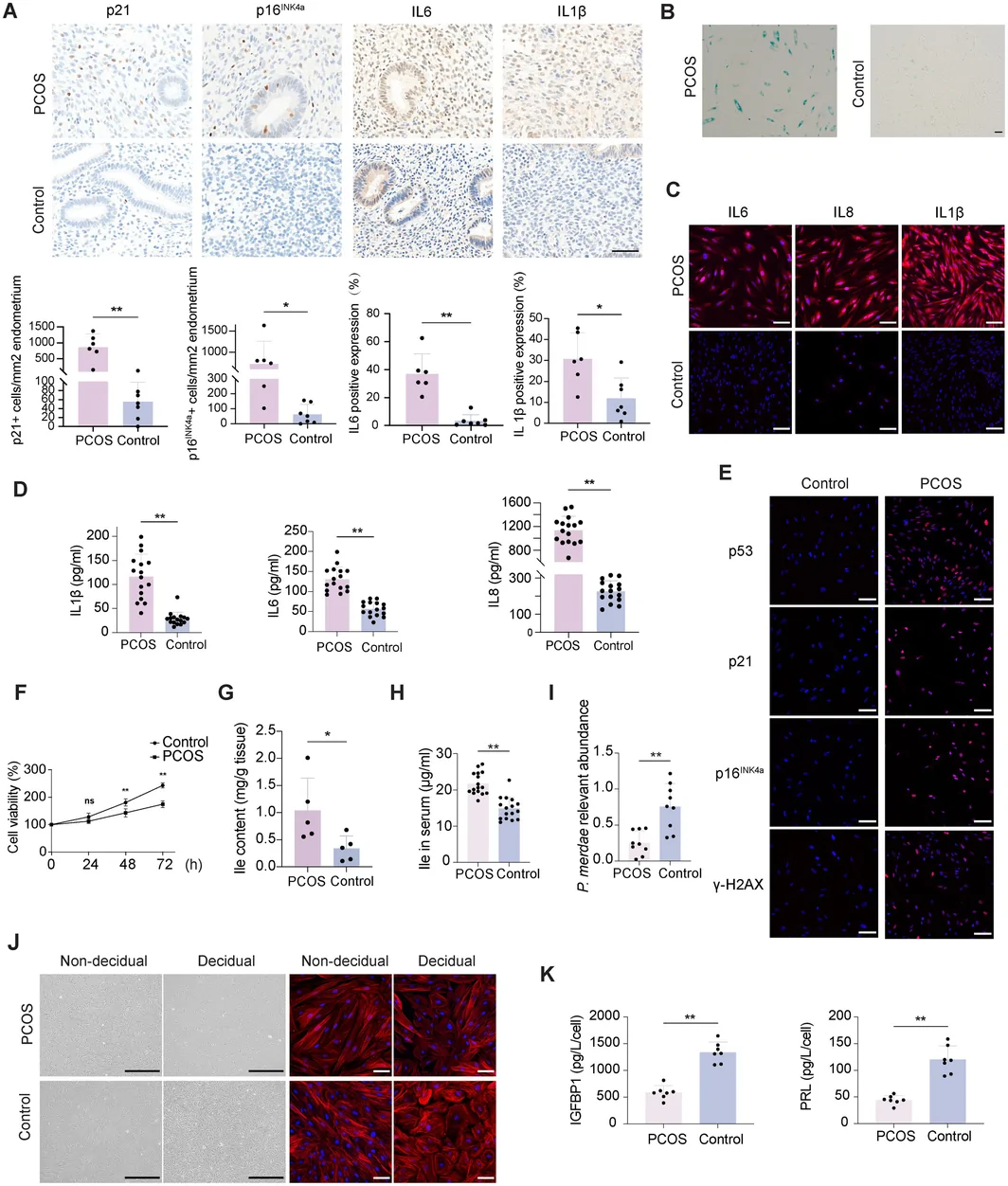
Senescence characteristics and impaired differentiation potential of ESCs in patients with PCOS. (A) Immunohistochemical staining of p21, p16^*INK4a*^, IL‐6, and IL‐1β in endometrial tissues. Scale bar, 50 μm. (B) SA‐β‐Gal activity in ESCs. Scale bar, 100 μm. (C) Immunofluorescence staining of SASPs. Scale bar, 100 μm. (D) Concentrations of IL‐6, IL‐1β and IL‐8 in ESC culture media evaluated by ELISA. (E) Immunofluorescence staining of γH2AX, p53, p21, and p16^INK4a^. Scale bar, 100 μm. (F) Cell viability of hESCs from PCOS or control determined by CCK‐8 assay. (G) Comparison of Ile content in endometrial tissues from PCOS and control groups. (H) Serum Ile level in PCOS and control groups. (I) Comparison of *P. merdae* abundance between PCOS and control groups. (J) Bright‐field (scale bar, 500 μm) and F‐Actin cytoskeleton (scale bar, 100 μm) images of ESCs after decidualisation induction. (K) Concentrations of PRL and IGFBP1 in ESC culture media evaluated by ELISA. *p < 0.05, **p < 0.01.

Functional assessment of decidualization capacity showed that PCOS ESCs largely failed to undergo the characteristic morphological shift from fibroblastic to epithelioid upon hormonal stimulation as control ESCs (Figure 2J). Therefore, PCOS ESCs secreted significantly lower levels of decidualization markers PRL and IGFBP1 than controls (Figure 2K).

### 3.4 Ile Induces Senescence and Decidualization Dysfunction in ESCs

To investigate the effects of Ile on endometrial function, primary ESCs were treated with increasing concentrations of Ile (5, 10, and 20 mM). Ile exposure induced a dose‐dependent increase in senescence markers, including SA‐β‐Gal level (Figure 3A) and intracellular ROS levels (Figure 3B). Ile treatment upregulated the DNA damage marker γ‐H2AX and SASP factors in a dose‐dependent manner (Figure 3C). Ile exposure also suppressed cell viability (Figure 3D) and induced cell cycle arrest (Figure 3E). Additionally, Ile treatment inhibited the secretion of decidualization markers PRL and IGFBP1 (Figure 3F,G). RNA sequencing of Ile‐treated versus untreated ESCs identified 246 upregulated and 284 downregulated genes (Figure 3H). GO and KEGG pathway analyses showed enrichment in chemokine signalling, PI3K‐Akt signalling, NOD‐like receptor signalling, and focal adhesion pathways (Figure 3I,J).

**FIGURE 3:**
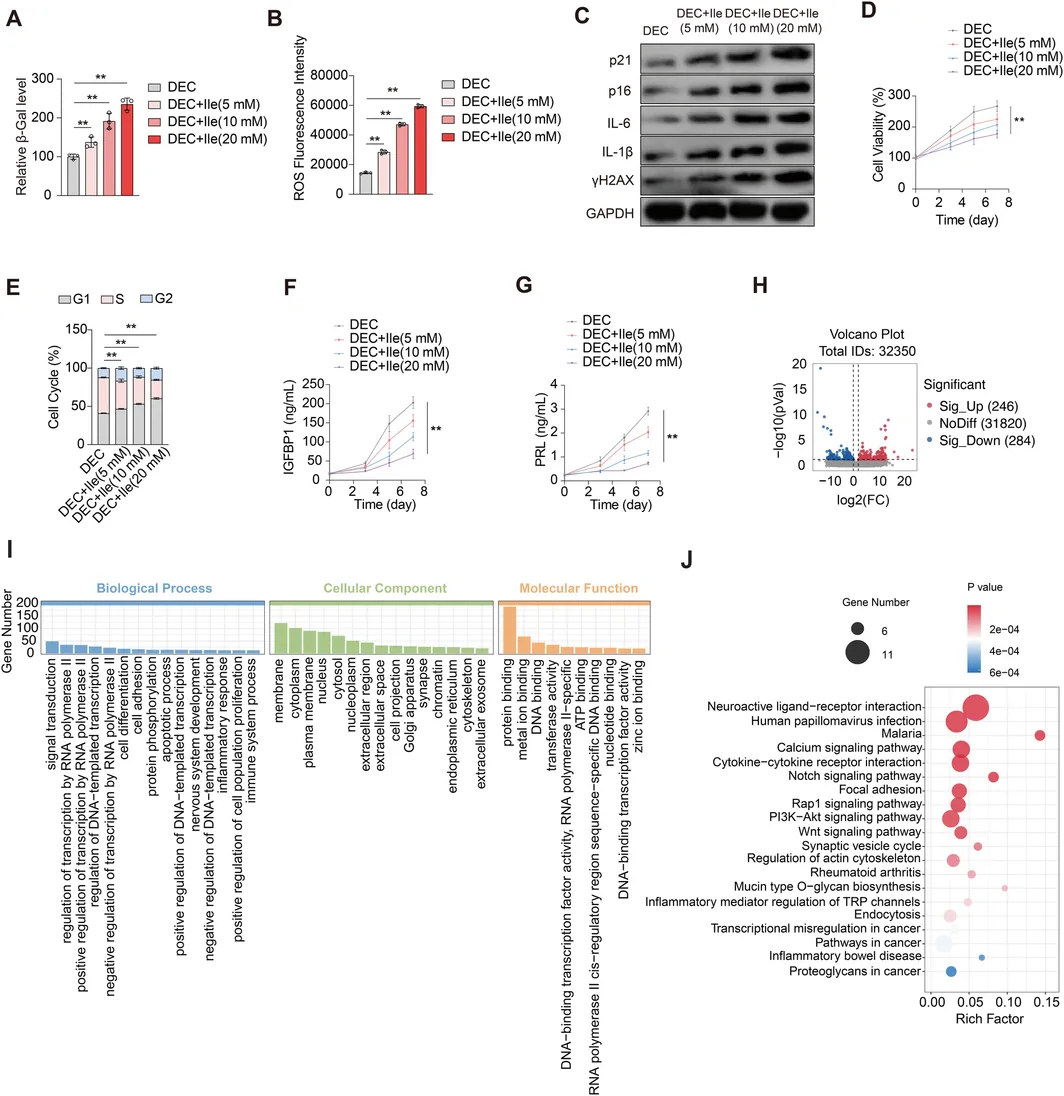
Exogenous isoleucine treatment induces senescence and impairs decidualisation capacity in decidualised endometrial cells (DECs). (A) SA‐β‐Gal activity in DECs. (B) Relative ROS levels in DECs. (C) Western blot analysis of p21, p16^*INK4a*^, IL‐1β, IL‐6, and γ‐H2AX in DECs treated with Ile. (D) Cell viability of DECs. (E) Cell cycle distribution in DECs. (F–G) Secretion of decidualisation markers. (H) Volcano plot of differentially expressed genes. (I) GO enrichment analysis. (J) KEGG pathway enrichment analysis. **p < 0.01.

### 3.5 *P. merdae* Intervention Ameliorates Metabolic and Reproductive Dysfunction in PCOS Rats

A well‐established rat model of PCOS was used to investigate the effects of *P. merdae* supplementation on metabolic dysfunction and reproductive outcomes (Figure 4A). Letrozole‐treated PCOS rats exhibited increased body weight and elevated serum Ile levels, which were significantly reduced by *P. merdae* supplementation (Figure 4B,C). Ovarian dysfunction was evident in PCOS rats, showing disrupted estrous cycles, increased follicular cysts, and elevated serum LH and T levels with an increased LH/FSH ratio (Figure 4D–I). Direct *P. merdae* supplementation or fecal microbiota transplantation (FMT) ameliorated these reproductive abnormalities, with improvements in estrous cyclicity, cyst formation, and hormonal imbalances. Additionally, *P. merdae* supplementation effectively reversed lipid accumulation in adipose tissue and hepatic steatosis (Figure S12A,B).

**FIGURE 4:**
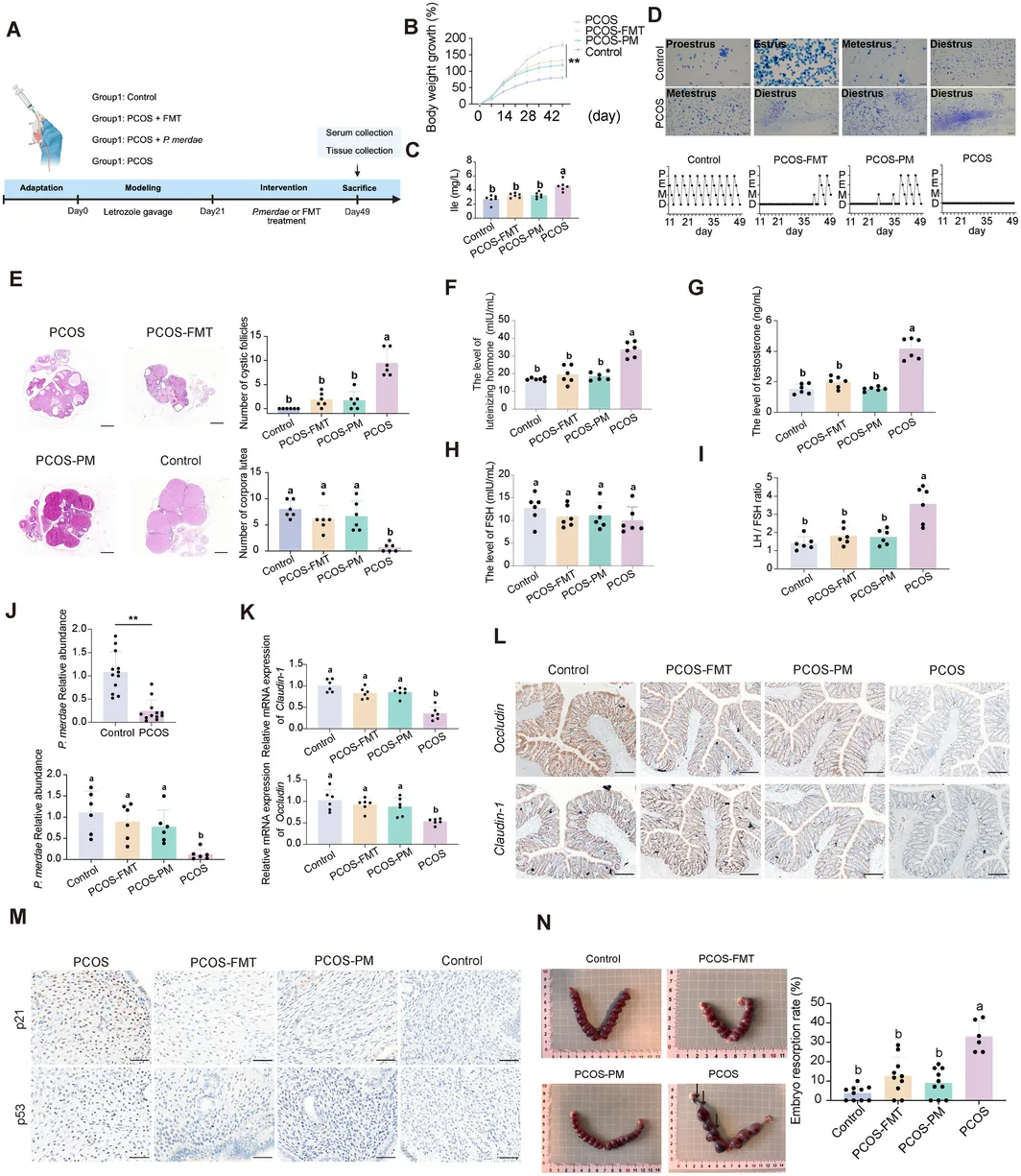
*P. merdae* treatment reverses PCOS‐associated metabolic dysfunction, endometrial senescence, and fetal resorption in rats. (A) Animal experimental design. (B) Body weight changes. (C) Serum Ile content. (D) Vaginal cytology. Representative images of vaginal smears from control and PCOS rats. Scale bar, 100 μm. The oestrous cycle charts show the daily vaginal cytology and oestrous stages for individual rats in the different experimental groups. P, E, M, and D represent the Pro‐oestrus, Oestrus, Metoestrus, and Dioestrus stages, respectively. (E) Ovarian histology (H&E staining) and follicular cyst quantification. Scale bar, 1000 μm. (F,I) Serum levels of LH, T, FSH, and the LH/FSH ratio in PCOS rats. (J) Relative abundance of *P. merdae* in faecal samples before and after treatment intervention. (K) Tight junction gene expression (*Claudin‐1* and *Occludin*) in colonic tissues. (L) Immunohistochemistry of tight junction proteins in colonic tissues. Scale bar, 200 μm. (M) Representative immunohistochemical staining of endometrial tissues for senescence markers P21 and P53. Scale bar, 50 μm. (N) Fetal resorption rates. The background board scale is in centimetres. Bars with different letters indicate statistically significant differences between groups (*p* < 0.05). Figure 4A was created by M.J. using BioRender.com (https://BioRender.com/bm6iazp).

Following successful *P. merdae* colonization in PCOS rat colons (Figure 4J), intestinal barrier integrity was significantly improved, as evidenced by enhanced expression of tight junction proteins *Claudin‐1* and *Occludin* in colonic tissue (Figure 4K,L). Furthermore, *P. merdae* supplementation attenuated elevated *p21* and *p53* expression in the PCOS endometrium (Figure 4M) and reduced fetal resorption rates (Figure 4N).

## 4 Discussion

### 4.1 Main Findings

This integrated human and animal study characterizes gut microbiota and metabolic disturbances associated with reproductive dysfunction in PCOS (Figure S13). We demonstrated that depletion of *P. merdae* in the gut microbiota aligned with elevated systemic and local endometrial levels of Ile, a branched‐chain amino acid previously implicated in metabolic disorders but not directly linked to reproductive dysfunction. This microbial‐metabolite perturbation was associated with cellular senescence in ESCs, and these features were collectively observed alongside elevated miscarriage risk in patients with PCOS.

Importantly, *P. merdae* supplementation in a letrozole‐induced PCOS rat model ameliorated several key features, producing multi‐system effects, restoring gut barrier integrity, reducing circulating and endometrial Ile concentrations, attenuating the senescent endometrial phenotype, and reducing fetal resorption rates. While these findings provide mechanistic insights, reduced fetal resorption in a rat model is not directly equivalent to improved pregnancy outcomes in women with PCOS, and clinical translation requires dedicated human studies.

### 4.2 Strengths and Limitations

Discovery‐driven multi‐omics approaches have been recognized as offering particular promise for clarifying the pathophysiology of PCOS, a heterogeneous syndrome for which no single unifying mechanistic hypothesis has been established yet [29]. Founded on this methodological framework, the present study provides novel evidence of an association between the gut microbiome–metabolite axis, specifically *P. merdae* depletion and Ile accumulation. Although the precise molecular mechanisms (e.g., specific enzymes, microbial competition, or host‐mediated effects) warrant further investigation, our findings help define priorities for future mechanistic and translational research regarding endometrial senescence and miscarriage in patients with PCOS.

We acknowledge the limitations of this study. Our control group comprised women with tubal factor infertility rather than truly healthy individuals. All participants (both PCOS and control groups) underwent IVF/ICSI treatment, which minimized confounding from differential treatment backgrounds. We carefully excluded tubal patients with severe hydrosalpinx or active pelvic inflammation. Nonetheless, future studies including true healthy controls would provide additional confirmation. Additionally, the small number of miscarriage events in this study precluded a reliable multivariable logistic regression analysis. Therefore, only unadjusted associations are reported. Future large‐scale studies are needed to confirm these findings after adjusting for relevant confounders.

### 4.3 Interpretation

Previous studies have reported depletion of the *Parabacteroides* genus in PCOS patients [30]. Our 16S rRNA sequencing findings corroborate this observation. Building upon these findings, we identified *P. merdae* , a specific species within the *Parabacteroides* genus, as a potential contributor to BCAA metabolic disturbances in PCOS. The role of *P. merdae* in BCAA metabolism is well documented through its *porA* gene‐mediated catabolic capacity [31], and its depletion in PCOS cohorts has been previously reported [32]. Given that young women with PCOS exhibit perturbations in redox homeostasis and lipophilic antioxidant capacity, the cardiovascular implications of *P. merdae* depletion and subsequent BCAA accumulation may extend beyond reproductive dysfunction that warrants further investigation [33]. Notably, while bacteria such as *Prevotella copri* and *Clostridium symbiosum* primarily synthesize BCAAs [34, 35, 36], *P. merdae* is functionally distinct in its capacity to catabolize BCAAs via *porA*‐mediated conversion of Leu, Ile, and Val into branched‐SCFAs [37], supporting further investigation of its supplementation in PCOS.

The finding that Ile induced ESC senescence in a dose‐dependent manner in vitro provides mechanistic insight into a potential pathway by which metabolic disturbances could contribute to reproductive dysfunction. This finding is supported by recent evidence showing that blocking BCAA accumulation can mitigate senescence in human bronchial epithelial cells [38] and that high‐Leu diets drive decidual stromal cell senescence and embryo loss in mice [25]. The concept of aberrant cellular senescence contributing to reproductive failure is consistent with emerging understanding in reproductive biology. While controlled senescence plays recognized physiological roles in decidualization [39], an imbalance in senescent decidual cell populations has been implicated in implantation failure and pregnancy loss [40]. Indeed, decidual stromal cells from patients with recurrent pregnancy loss demonstrate persistent proinflammatory SASP profiles [41, 42, 43], and limiting senescent cell accumulation in the decidua can prevent spontaneous abortion.

Our findings that *P. merdae* supplementation reverses PCOS phenotype in vivo may inform future therapeutic strategies aiming at modifying gut microbial and metabolic profiles in women with PCOS prior to conception. The demonstration that *P. merdae* supplementation reduced fetal resorption rates and attenuated endocrine abnormalities and endometrial senescence markers in a letrozole‐induced PCOS rat model suggests that microbiota‐directed interventions may represent a biologically plausible strategy, although the limitations of this induced animal model must be acknowledged and the clinical relevance to human PCOS remains to be established. Mechanistically, depletion of *P. merdae* in PCOS patients may therefore contribute to reduced BCAA catabolism and subsequent systemic Ile accumulation. Emerging evidence suggests that BCAA accumulation may activate mTORC1 signalling and contribute to human bronchial epithelial cell senescence through upregulation of BCAA transporters (SLC6A14, SLC6A15) and downregulation of BCAA catabolic enzyme BCAT1. Our in vitro data show that Ile treatment induced an ESC senescence phenotype characterized by increased *p53*, *p21*, *p16* ^*INK4a*^, ROS generation, and IL‐1β, IL‐6 and IL‐8 expression. The SASP factor concentrations in control ESC conditioned media were broadly consistent with previously reported baseline levels in primary human endometrial stromal cell cultures [44, 45, 46], suggesting that the elevations observed in PCOS‐derived and Ile‐treated ESCs represent a biologically meaningful departure from physiological norms. Notably, this senescence phenotype was accompanied by impaired cellular function, including reduced proliferative capacity and diminished secretion of key decidualization markers [47, 48].

Intriguingly, given that aberrant mTOR activation has been implicated in premature ovarian follicle activation and depletion [49], and that Leu may influence primordial follicle proliferation and differentiation [50], modulation of BCAA metabolism through *P. merdae* supplementation may have broader implications for ovarian function and oocyte quality, although this was not directly examined in the present study. Beyond probiotic interventions, the observation that Ile induced senescence and SASP in our cellular models raises the possibility that dietary modulation of BCAA intake warrants future investigation. Although high‐protein hypocaloric diets have shown metabolic benefits in PCOS women [51], direct evidence linking dietary BCAA modulation to reproductive outcomes remains lacking.

## 5 Conclusions

This study supports the potential existence of a gut microbiome–metabolite axis as a putative mechanistic link in PCOS‐related reproductive dysfunction. By integrating multi‐omics analyses with functional validation, our findings indicate that gut dysbiosis and concurrent systemic and local BCAA accumulation are associated with impaired endometrial function and elevated risk of miscarriage. Importantly, *P. merdae* supplementation ameliorated several of these abnormalities and reduced fetal resorption rates in PCOS rats. These findings provide a novel mechanistic framework outlining PCOS‐related reproductive dysfunction and further investigation of microbiota‐based interventions as a potential approach for improving reproductive outcomes.

## Author Contributions

M.J.and X.C.: contributed equally to this work. A.L.: conceived the overall research framework and direction and supervised the project. Sample collection was conducted by M.J., X.C., H.F., and M.J. Follow‐up data collection was carried out by X.C. Data analysis was performed by M.J., X.C. and A.L. M.J. and X.C. conducted the cell experiments and drafted the manuscript. X.Z., X.J., A.L., W.G., N.H., and Y.J. revised the initial draft. All authors have read and agreed to the published version of the manuscript.

## Funding

This study was supported by the Natural Science Foundation of Xinjiang Uygur Autonomous Region (No. 2022D01C592) and the Hangzhou Joint Fund of the Zhejiang Provincial Natural Science Foundation of China (No. LHZSD24H290001).

## Ethics Statement

This study was approved by the Ethics Committee of Women's Hospital, School of Medicine, Zhejiang University (approval number IRB‐20220168‐R, 2022), and animal experiments were approved by the Laboratory Animal Welfare and Ethics Committee of the same institution (approval number AE 20250023, 2025).

## Conflicts of Interest

The authors declare no conflicts of interest.

## Supporting information


**Table S1:** Clinical characteristics of PCOS patients and controls in the multi‐omics study.
**Table S2:** Clinical characteristics of participants in the 16S gene sequencing.
**Table S3:** Clinical characteristics of participants in the metagenomics and untargeted metabolomics study.
**Table S4:** Clinical characteristics of participants in the targeted metabolomics.
**Table S5:** Clinical characteristics of women with PCOS and controls undergoing endometrial sampling.
**Table S6:** KEGG functional modules differentially enriched in gut microbiota between PCOS patients and Controls.
**Table S7:** Clinical pregnancy outcomes and treatment types among women with PCOS and controls during follow‐up.
**Figure S1:** Distribution of PCOS phenotypes in the study cohort (*n* = 110).
**Figure S2:** 16S rRNA sequencing reveals microbiome differences between PCOS patients (*n* = 30) and Controls (*n* = 30).
**Figure S3:** Correlations between key gut bacterial genera identified by 16S rRNA sequencing and clinical features in all participants.
**Figure S4:** Microbial community differences between PCOS (*n* = 40) and Control (*n* = 40) revealed on metagenomic sequencing.
**Figure S5:** Association of metagenomic‐identified key gut bacterial species with clinical features in all participants.
**Figure S6:** Identification of amino acids and SCFAs as potential molecular markers and their associations with PCOS clinical features.
**Figure S7:** Receiver operating characteristic curves for serum isoleucine and P. merdae abundance in predicting miscarriage.
**Figure S8:** Serum isoleucine levels and P. merdae abundance across PCOS phenotypes and Control group.
**Figure S9:** Transcriptional profile analysis and functional enrichment of differentially expressed genes.
**Figure S10:** Integrative analysis of endometrial senescence in PCOS patients.
**Figure S11:** Correlations between gut microbiota, serum branched‐chain amino acids, and SASP factors.
**Figure S12:** Effects of P. merdae supplementation on adipose tissue and liver in PCOS rats.
**Figure S13:** Summary of study findings illustrating the association between gut microbiota dysbiosis, isoleucine accumulation, endometrial senescence, and miscarriage risk in women with PCOS.

## Data Availability

The data that support the findings of this study are available from the corresponding author upon reasonable request.
