# Supplementary material for: Microbial and Metabolic Correlates of Endometrial Dysfunction in Polycystic Ovary Syndrome: A Translational Study
Source: BJOG. 2026 May 19;133(12):2092–102. doi: 10.1111/1471-0528.70266 (PMC13659357; doi:10.1111/1471-0528.70266)
Supplement: Supplementary file 1 [file BJO-133-2092-s001.docx]

**SI Appendix**

**Microbial and Metabolic Correlates of Endometrial Dysfunction in Polycystic Ovary Syndrome: A Translational Study**

Mengyu Jing^1^, Xi Chen^1^, Meiyan Jiang, Huiya Fang, Xiaoming Zhu, Xuejing Jin, Yonghui Jiao, Ningning Hou, Weiwei Gong, Aixia Liu*

^1^Mengyu Jing and Xi Chen contributed equally to this article.

^*^Corresponding Author. liuaixia@zju.edu.cn (A. Liu).

This file includes:

Supplementary Methods.

Table S1 to 7.

Figure S1 to 13.

**Content**

|  | Title | Page |
| --- | --- | --- |
| **Supplementary Methods** | Supplementary Methods | 3-6 |
| **Table S1** | Clinical characteristics of PCOS patients and controls in the multi-omics study. | 7 |
| **Table S2** | Clinical characteristics of participants in the 16S gene sequencing. | 8 |
| **Table S3** | Clinical characteristics of participants in the metagenomics and untargeted metabolomics study. | 9 |
| **Table S4** | Clinical characteristics of participants in the targeted metabolomics. | 10 |
| **Table S5** | Clinical characteristics of women with PCOS and controls undergoing endometrial sampling. | 11 |
| **Table S6** | KEGG functional modules differentially enriched in gut microbiota between PCOS patients and Controls. | 12-13 |
| **Table S7** | Clinical pregnancy outcomes and treatment types among women with PCOS and controls during follow-up. | 13 |
| **Figure S1** | Distribution of PCOS phenotypes in the study cohort (n = 110). | 14 |
| **Figure S2** | 16S rRNA sequencing reveals microbiome differences between PCOS patients (n = 30) and Controls (n = 30). | 15 |
| **Figure S3** | Correlations between key gut bacterial genera identified by 16S rRNA sequencing and clinical features in all participants. | 16 |
| **Figure S4** | Microbial community differences between PCOS (n = 40) and Control (n = 40) revealed on metagenomic sequencing. | 17 |
| **Figure S5** | Association of metagenomic-identified key gut bacterial species with clinical features in all participants. | 18 |
| **Figure S6** | Identification of amino acids and SCFAs as potential molecular markers and their associations with PCOS clinical features. | 19 |
| **Figure S7** | Receiver operating characteristic curves for serum isoleucine and P. merdae abundance in predicting miscarriage. | 20 |
| **Figure S8** | Serum isoleucine levels and *P. merdae* abundance across PCOS phenotypes and Control group. | 20 |
| **Figure S9** | Transcriptional profile analysis and functional enrichment of differentially expressed genes. | 21 |
| **Figure S10** | Integrative analysis of endometrial senescence in PCOS patients. | 21 |
| **Figure S11** | Correlations between gut microbiota, serum branched-chain amino acids, and SASP factors. | 22 |
| **Figure S12** | Effects of *P. merdae* supplementation on adipose tissue and liver in PCOS rats. | 23 |
| **Figure S13** | Summary of study findings illustrating the association between gut microbiota dysbiosis, isoleucine accumulation, endometrial senescence, and miscarriage risk in women with PCOS. | 24 |

**Supplementary Methods**

**1.1 Multi-omics profiling of gut microbiota and serum metabolites**

Genomic DNA was extracted using the PureLink™ Stool Genomic DNA Kit (ThermoFisher Scientific Inc., United States). For 16S rRNA gene sequencing, 60 fecal samples were analyzed (30 PCOS vs. 30 controls). Additionally, metagenomic libraries were prepared from fecal DNA samples (40 PCOS vs. 40 controls) and sequenced on a NovaSeq 6000 using PE150 chemistry.

Untargeted serum metabolomics was performed in the cohort subjected to metagenomic analysis using liquid chromatography-tandem mass spectrometry (LC-MS/MS). Metabolites were extracted using organic solvent precipitation, with quality control samples included to ensure technical reproducibility. Targeted quantification of amino acids and SCFAs was conducted on serum samples (60 PCOS vs. 60 controls).

**1.2 Primary Endometrial Stromal Cells Isolation and Culture**

Endometrial samples were collected from a subset of 32 participants (PCOS vs. Control, n = 16 vs. 16) drawn from the overall cohort during the proliferative phase via hysteroscopy. Primary ESCs were induced to decidualize in vitro using a hormonal cocktail containing 10 nM 17β-estradiol (Sigma, 2758), 1 μM medroxyprogesterone acetate (MCE, HY-B0469), and 0.5 mM cAMP (MCE, HY-B1511) for the indicated durations. To investigate the effects of isoleucine (Ile), cells were exposed to decidualization inducers with or without Ile supplementation at varying concentrations (5, 10, and 20 mM; MCE, HY-N0771).

**1.3 Animal Models and Treatments**

All animal experiments were approved by the Institutional Laboratory Animal Care and Use Committee (AE 20250023). Sixty-four 5-week-old female Sprague-Dawley (SD) rats were acclimatized for 1 week and then divided into two groups: Control (receiving CMC gavage) and PCOS (receiving letrozole gavage at 1 mg/kg once daily combined with a high-fat diet for 21 days). The PCOS group was further subdivided into three groups: PCOS (saline), PCOS-FMT (receiving 200 μL fecal bacterial suspension), and PCOS-PM (receiving 200 μL saline containing *P. merdae* at 2 × 10⁸ CFU/mL) [1]. After the 4-week intervention, blood, ovarian, and uterine tissue samples were collected for subsequent experiments.

**1.4 Immunohistochemistry**

Paraffin-embedded tissue sections (4 μm) were deparaffinized, rehydrated, and subjected to antigen retrieval. Sections were incubated overnight at 4°C with primary antibodies, followed by HRP-conjugated secondary antibody (Affinity, S0001) for 30 min. Signal development was performed using DAB chromogen (Biosharp, BL732A, China).

**1.5 Enzyme-Linked Immunosorbent Assay**

Conditioned media from ESC cultures were collected to measure pro-inflammatory cytokines and decidualisation markers using commercial ELISA kits (Jianglai, Shanghai, China; Elabscience, Wuhan, China), following the manufacturers’ instructions. Absorbance was measured at 450 nm using a multi-mode microplate reader (SpectraMAX iD5®, USA).

**1.6 Flow Cytometry**

Fixed cells were stained with propidium iodide solution (Beyotime, C1052) and analyzed for DNA content (Beckman Cytoflex S, USA). For intracellular reactive oxygen species (ROS) detection, cells were collected, stained with 10 µM 2',7'-dichlorodihydrofluorescein diacetate (DCFH-DA; Beyotime, S0033) in DMEM at 37°C for 30 min in the dark, and analyzed by flow cytometry.

**1.7 Western Blotting**

Membranes were blocked with 5% nonfat milk in TBST and incubated overnight at 4°C with primary antibodies against *p21* (Abcam, ab109520), *p16* (Abcam, ab270058), *IL-6* (Abcam, ab233551), *IL-1β* (Abcam, ab283818), *γ-H2AX* (Cell Signaling Technology, #9718), or *GAPDH* (Abcam, ab8245). Protein bands were visualized using ECL reagent (BIO-RAD, 170-5061, Hercules, USA) and imaged.

**1.8 Quantitative Real-Time PCR**

Total RNA was extracted using Trizol reagent (Takara Bio, Japan) following the manufacturer’s procedure. Primer sequences used were as follows: *β-actin* forward (5'-CTAGGCACCAGGGTGTGATG-3') and reverse (5'-AGGTCTCAAACATGATCTGGGT-3'); *Occludin* forward (5'-GGGGCGCAGCAGGTCT-3') and reverse (5'-GCCTGTAAGGAGGTGGACTC-3'); *Claudin-1* forward (5'-TGGGGCTGATCGCAATCTTT-3') and reverse (5'-CACTAATGTCGCCAGACCTGA-3'); bacterial *16S rRNA* forward (5'-WACGCGARGAACCTTACC-3') and reverse (5'-TGACGGGCGGTGWGTAC-3'); *P. merdae*-specific forward (5'-AGTGTGTTTGAGGTAGGCGG-3') and reverse (5'-ACGCTTTCGCTGTAGAGCTT-3').

**1.9 Statistical Analysis**

Statistical analysis was performed using GraphPad Prism (10.0, USA). For comparisons between two independent groups, normality and homogeneity of variance were first assessed. If the data were approximately normally distributed and variance homogeneity was satisfied, Student’s t-test was applied; if the data were approximately normally distributed but variances were unequal, Welch’s t-test was used; otherwise, the Mann–Whitney U test was performed. Multiple group comparisons were analyzed using one-way ANOVA followed by Tukey test. Specifically, differences in microbial community composition were assessed using both unsupervised and supervised methods. Unsupervised analyses included principal component analysis (PCoA) based on Euclidean distance of normalized data and principal coordinate analysis (PCA) based on Bray-Curtis distances, with statistical significance tested by permutational multivariate analysis of variance. Supervised analysis was performed using partial least squares discriminant analysis with 7-fold cross-validation to maximize group separation. Random forest analysis was performed to identify bacterial genus with the highest discriminatory power between PCOS patients and control women. Variable importance was ranked by the mean decrease Gini index. Data are presented as mean ± standard deviation or median (interquartile range) as appropriate. Differences were considered statistically significant at *p* < 0.05. Correlations between variables were assessed using Spearman's rank correlation coefficient.

**References**

[1]	Zhang DY, Li D, Chen SJ, Zhang LJ, Zhu XL, Chen FD, et al. Bacteroides uniformis-generated hexadecanedioic acid ameliorates metabolic-associated fatty liver disease. Gut Microbes. 2025;17(1):2508433.

**Table S1. Clinical characteristics of PCOS patients and controls in the multi-omics study.**

|  | PCOS ( n =110 ) | Control ( n =110 ) | *p* value |
| --- | --- | --- | --- |
| Age (year) | 29.00 ( 27.25 , 32.00 ) | 29.00 ( 28.00 , 31.00 ) | 0.739 |
| BMI | 22.48 ( 20.65 , 24.56 ) | 21.77 ( 20.23 , 24.03 ) | 0.233 |
| AMH (ng/mL) | 8.14 ( 6.26 , 12.50 ) | 2.83 ( 1.94 , 4.72 ) | < 0.001 |
| Estradiol (pmol/L) | 147.20 ( 116.00 , 179.00 ) | 136.50 ( 113.00 , 178.75 ) | 0.290 |
| Homocysteine (μmol/L) | 8.10 ( 7.30 , 9.78 ) | 6.85 ( 6.12 , 7.90) | < 0.001 |
| FSH (IU/L) | 5.88 ( 5.10 , 6.65 ) | 6.12 ( 5.31 , 6.91 ) | 0.094 |
| LH (IU/L) | 11.70 ( 9.37 , 15.80 ) | 5.24 ( 4.06 , 6.36 ) | < 0.001 |
| LH/FSH ratio | 2.08 ( 1.65 , 2.59 ) | 0.81 ( 0.61 , 1.10 ) | < 0.001 |
| Testosterone (nmol/L) | 1.40 ( 1.00 , 1.80 ) | 0.60 ( 0.40 , 1.00 ) | < 0.001 |
| Progesterone (nmol/L) | 0.87 ( 0.64 , 1.23 ) | 0.99 ( 0.67 , 1.49 ) | 0.291 |
| Prolactin (ng/mL) | 15.45 ( 11.78 , 20.65 ) | 16.60 ( 12.07 , 22.30 ) | 0.512 |
| TSH (mIU/L) | 1.68 ( 1.29 , 2.15 ) | 1.46 ( 1.16 , 1.99 ) | 0.106 |
| Low-density lipoprotein (mmol/L) | 2.88 ( 2.42 , 3.43 ) | 2.68 ( 2.28 , 3.18 ) | 0.028 |
| High-density lipoprotein (mmol/L) | 1.46 ( 1.20 , 1.82 ) | 1.41 ( 1.22 , 1.56 ) | 0.063 |
| Triglyceride (mmol/L) | 1.69 ( 1.18 , 2.30 ) | 0.94 ( 0.73 , 1.31 ) | < 0.001 |
| Total cholesterol (mmol/L) | 4.84 ( 4.08 , 5.79 ) | 4.54 ( 3.91 , 4.98 ) | 0.003 |
| Fasting insulin (μU/mL) | 9.90 (6.93, 12.78) | 7.30 (5.10, 11.80) | 0.003 |
| Fasting blood glucose (mmol/L) | 4.87 ± 0.38 | 4.95 ± 0.36 | 0.103 |
| HOMA-IR | 2.00 (1.21, 2.86) | 1.58 (1.02, 2.51) | 0.019 |

Abbreviation: BMI：Body Mass Index；AMH：Anti-Müllerian Hormone；FSH：Follicle-Stimulating Hormone；LH：Luteinizing Hormone；TSH：Thyroid-Stimulating Hormone.

**Table S2. Clinical characteristics of participants in the 16S rRNA gene sequencing.**

|  | PCOS ( n = 30 ) | Control ( n = 30 ) | *p* value |
| --- | --- | --- | --- |
| Age (year) | 29.17 ± 2.42 | 29.20 ± 2.91 | 0.962 |
| BMI | 22.53 ± 2.35 | 22.22 ± 2.48 | 0.620 |
| AMH (ng/mL) | 7.53 ( 6.00 , 13.00 ) | 3.17 ( 1.93 , 4.38 ) | < 0.001 |
| Estradiol (pmol/L) | 146.60 ( 105.25 , 186.00 ) | 134.40 ( 103.00 , 174.45 ) | 0.371 |
| Homocysteine (μmol/L) | 8.65 ± 1.95 | 6.73 ± 1.59 | 0.000 |
| FSH (IU/L) | 5.90 ± 1.40 | 6.33 ± 1.43 | 0.242 |
| LH (IU/L) | 12.23 ± 4.17 | 4.68 ± 1.96 | < 0.001 |
| LH/FSH ratio | 2.24 ( 1.75 , 2.41 ) | 0.67 ( 0.53 , 0.94 ) | < 0.001 |
| Testosterone (nmol/L) | 1.30 ( 0.92 , 1.80 ) | 0.60 ( 0.40 , 1.00 ) | < 0.001 |
| Progesterone (nmol/L) | 0.84 ( 0.75 , 1.09 ) | 0.87 ( 0.59 , 1.17 ) | 0.717 |
| Prolactin (ng/mL) | 17.54 ± 6.55 | 17.29 ± 9.88 | 0.910 |
| TSH (mIU/L) | 1.74 ( 1.37 , 1.92 ) | 1.42 ( 1.10 , 1.85 ) | 0.092 |
| Low-density lipoprotein (mmol/L) | 2.84 ( 2.20 , 3.25 ) | 2.55 ( 2.20 , 2.84 ) | 0.193 |
| High-density lipoprotein (mmol/L) | 1.50 ± 0.32 | 1.38 ± 0.25 | 0.114 |
| Triglyceride (mmol/L) | 1.79 ( 1.28 , 2.61 ) | 1.02 ( 0.79 , 1.47 ) | < 0.001 |
| Total cholesterol (mmol/L) | 4.90 ( 3.94 , 5.79 ) | 4.22 ( 3.86 , 4.92 ) | 0.080 |
| Fasting insulin (μU/mL) | 11.05 ( 7.78 , 13.88 ) | 9.85 ( 5.03 , 12.55 ) | 0.261 |
| Fasting blood glucose (mmol/L) | 4.90 ± 0.39 | 4.95 ± 0.37 | 0.582 |
| HOMA-IR | 2.39 ( 1.69 , 3.16 ) | 2.19 ( 1.02 , 2.86 ) | 0.307 |

Abbreviation: BMI：Body Mass Index；AMH：Anti-Müllerian Hormone；FSH：Follicle-Stimulating Hormone；LH：Luteinizing Hormone；TSH：Thyroid-Stimulating Hormone.

**Table S3. Clinical characteristics of participants in the metagenomics and untargeted metabolomics study.**

|  | PCOS ( n = 40 ) | Control ( n = 40 ) | *p* value |
| --- | --- | --- | --- |
| Age (year) | 29.00 ( 26.75 , 32.00 ) | 29.50 ( 28.00 , 31.00 ) | 0.961 |
| BMI | 23.34 ( 20.70 , 25.64 ) | 21.28 ( 19.48 , 23.82 ) | 0.074 |
| AMH (ng/mL) | 8.19 ( 6.12 , 15.50 ) | 2.66 ( 2.08 , 4.51 ) | < 0.001 |
| Estradiol (pmol/L) | 157.00 ( 117.50 , 185.50 ) | 145.00 ( 121.00 , 181.55 ) | 0.776 |
| Homocysteine (μmol/L) | 8.00 ( 7.38 , 9.05 ) | 6.75 ( 6.18 , 8.00 ) | 0.002 |
| FSH (IU/L) | 6.00 ( 4.92 , 6.75 ) | 6.28 ( 5.88 , 7.04 ) | 0.108 |
| LH (IU/L) | 13.33 ± 6.11 | 5.61 ± 1.64 | < 0.001 |
| LH/FSH ratio | 2.23 ± 0.96 | 0.89 ± 0.28 | < 0.001 |
| Testosterone (nmol/L) | 1.40 ( 1.00 , 1.83 ) | 0.60 ( 0.30 , 0.95 ) | < 0.001 |
| Progesterone (nmol/L) | 0.69 ( 0.56 , 1.08 ) | 1.01 ( 0.66 , 1.54 ) | 0.051 |
| Prolactin (ng/mL) | 16.91 ± 7.27 | 20.01 ± 7.26 | 0.071 |
| TSH (mIU/L) | 1.49 ( 1.23 , 2.21 ) | 1.44 ( 1.10 , 2.02 ) | 0.548 |
| Low-density lipoprotein (mmol/L) | 2.64 ( 2.4 0, 3.11 ) | 2.80 ( 2.32 , 3.47 ) | 0.844 |
| High-density lipoprotein (mmol/L) | 1.47 ± 0.41 | 1.43 ± 0.24 | 0.585 |
| Triglyceride (mmol/L) | 1.62 ( 1.02 , 2.02 ) | 0.84 ( 0.70 , 1.10 ) | < 0.001 |
| Total cholesterol (mmol/L) | 4.65 ( 4.06 , 5.13 ) | 4.58 ( 3.90 , 5.45 ) | 0.557 |
| Fasting insulin (μU/mL) | 10.60 ( 7.00 , 12.40 ) | 7.25 ( 5.00 , 8.90 ) | 0.006 |
| Fasting blood glucose (mmol/L) | 4.83 ± 0.39 | 4.91 ± 0.30 | 0.270 |
| HOMA-IR | 2.18 ( 1.40 , 2.85 ) | 1.58 ( 1.09 , 1.97 ) | 0.027 |

Abbreviation: BMI：Body Mass Index；AMH：Anti-Müllerian Hormone；FSH：Follicle-Stimulating Hormone；LH：Luteinizing Hormone；TSH：Thyroid-Stimulating Hormone.

**Table S4. Clinical characteristics of participants in the targeted metabolomics.**

|  | PCOS ( n = 60 ) | Control ( n = 60 ) | *p* value |
| --- | --- | --- | --- |
| Age (year) | 31.00 ( 28.00 , 32.00 ) | 29.50 ( 28.00 , 32.00 ) | 0.554 |
| BMI | 22.39 ( 20.37 , 24.01 ) | 21.48 ( 19.72 , 23.71 ) | 0.337 |
| AMH (ng/mL) | 8.45 ( 6.24 , 11.43 ) | 2.83 ( 2.14 , 4.97 ) | < 0.001 |
| Estradiol (pmol/L) | 149.50 ( 118.15 , 175.25 ) | 134.50 ( 115.75 , 168.00 ) | 0.334 |
| Homocysteine (μmol/L) | 8.05 ( 7.30 , 9.80 ) | 7.10 ( 6.20 , 8.00 ) | < 0.001 |
| FSH (IU/L) | 5.93 ( 5.08 , 6.62 ) | 5.88 ( 5.25 , 6.53 ) | 0.852 |
| LH (IU/L) | 11.60 ( 9.38 , 16.12 ) | 5.33 ( 4.08 , 6.40 ) | < 0.001 |
| LH/FSH ratio | 2.09 ( 1.69 , 2.62 ) | 0.83 ( 0.66 , 1.12 ) | < 0.001 |
| Testosterone (nmol/L) | 1.40 ( 1.10 , 1.90 ) | 0.60 ( 0.40 , 0.92 ) | < 0.001 |
| Progesterone (nmol/L) | 1.00 ( 0.66 , 1.28 ) | 1.11 ( 0.81 , 1.64 ) | 0.104 |
| Prolactin (ng/mL) | 13.90 ( 11.40 , 20.17 ) | 14.80 ( 11.85 , 22.35 ) | 0.336 |
| TSH (mIU/L) | 1.52 ( 1.23 , 2.10 ) | 1.54 ( 1.25 , 1.95 ) | 0.688 |
| Low-density lipoprotein (mmol/L) | 3.09 ± 0.88 | 2.77 ± 0.55 | 0.018 |
| High-density lipoprotein (mmol/L) | 1.48 ( 1.23 , 1.86 ) | 1.42 ( 1.22 , 1.58 ) | 0.082 |
| Triglyceride (mmol/L) | 1.64 ( 1.11 , 2.25 ) | 0.89 ( 0.70 , 1.32 ) | < 0.001 |
| Total cholesterol (mmol/L) | 5.11 ± 1.31 | 4.55 ± 0.73 | 0.005 |
| Fasting insulin (μU/mL) | 7.75 ( 5.62 , 11.57 ) | 6.55 ( 5.10 , 8.73 ) | 0.035 |
| Fasting blood glucose (mmol/L) | 4.86 ± 0.38 | 4.98 ± 0.40 | 0.088 |
| HOMA-IR | 1.76 ( 1.19 , 2.63 ) | 1.46 ( 1.10 , 1.90 ) | 0.085 |

Abbreviation: BMI：Body Mass Index；AMH：Anti-Müllerian Hormone；FSH：Follicle-Stimulating Hormone；LH：Luteinizing Hormone；TSH：Thyroid-Stimulating Hormone.

**Table S5. Clinical characteristics of women with PCOS and controls undergoing endometrial sampling.**

|  | PCOS ( n = 16 ) | Control ( n = 16 ) | *p* value |
| --- | --- | --- | --- |
| Age (year) | 30.00 ( 28.75 , 32.00 ) | 30.00 ( 27.50 , 31.25 ) | 0.518 |
| BMI | 22.19 ( 21.04 , 23.71 ) | 22.46 ( 20.53 , 23.55 ) | 0.777 |
| AMH (ng/mL) | 3.26 ± 1.58 | 10.95 ± 4.74 | < 0.001 |
| Estradiol (pmol/L) | 134.61 ± 42.01 | 162.56 ± 37.36 | 0.056 |
| Homocysteine (μmol/L) | 6.62 ± 1.52 | 8.16 ± 1.62 | 0.010 |
| FSH (IU/L) | 5.86 ± 1.03 | 6.40 ± 1.43 | 0.230 |
| LH (IU/L) | 4.84 ( 4.03 , 6.25 ) | 12.25 ( 10.45 , 16.20 ) | < 0.001 |
| LH/FSH ratio | 0.77 ( 0.70 , 1.00 ) | 2.06 ( 1.64 , 2.36 ) | < 0.001 |
| Testosterone (nmol/L) | 0.40 ( 0.30 , 0.95 ) | 1.30 ( 0.98 , 1.70 ) | 0.001 |
| Progesterone (nmol/L) | 1.39 ( 0.99 , 1.66 ) | 1.08 ( 0.62 , 1.44 ) | 0.459 |
| Prolactin (ng/mL) | 17.10 ( 11.62 , 21.35 ) | 13.40 ( 10.98 , 18.10 ) | 0.638 |
| TSH (mIU/L) | 1.45 ( 1.22 , 1.79 ) | 1.61 ( 1.22 , 2.02 ) | 0.462 |
| Low-density lipoprotein (mmol/L) | 2.72 ± 0.55 | 3.31 ± 1.22 | 0.094 |
| High-density lipoprotein (mmol/L) | 1.44 ( 1.22 , 1.47 ) | 1.56 ( 1.23 , 1.89 ) | 0.207 |
| Triglyceride (mmol/L) | 0.83 ± 0.37 | 1.67 ± 0.67 | < 0.001 |
| Total cholesterol (mmol/L) | 4.48 ± 0.72 | 5.12 ± 1.85 | 0.213 |
| Fasting insulin (μU/mL) | 6.61 ± 1.89 | 10.04 ± 3.75 | 0.004 |
| Fasting blood glucose (mmol/L) | 4.93 ± 0.42 | 4.88 ± 0.44 | 0.765 |
| HOMA-IR | 1.46 ± 0.47 | 2.21 ± 0.92 | 0.008 |

Abbreviation: BMI：Body Mass Index；AMH：Anti-Müllerian Hormone；FSH：Follicle-Stimulating Hormone；LH：Luteinizing Hormone；TSH：Thyroid-Stimulating Hormone.

**Table S6. KEGG functional modules differentially enriched in gut microbiota between PCOS patients and Controls.**

| ID | Description | Report score | Z score | *p* value |
| --- | --- | --- | --- | --- |
| map00680 | Methane metabolism | -8.722 | -10.457 | < 0.001 |
| map02060 | Phosphotransferase system (PTS) | 7.685 | 8.736 | < 0.001 |
| map05171 | Coronavirus disease - COVID-19 | -6.081 | -7.141 | < 0.001 |
| map03010 | Ribosome | -5.867 | -7.096 | < 0.001 |
| map03010 | Ribosome | -5.867 | -7.096 | < 0.001 |
| map00860 | Porphyrin metabolism | 4.932 | 5.361 | < 0.001 |
| map03020 | RNA polymerase | -4.846 | -5.810 | < 0.001 |
| map02040 | Flagellar assembly | 4.669 | 5.088 | < 0.001 |
| map01200 | Carbon metabolism | -3.839 | -4.881 | < 0.001 |
| map01230 | Biosynthesis of amino acids | 3.582 | 3.645 | < 0.001 |
| map03030 | DNA replication | -3.547 | -4.372 | 0.001 |
| map00052 | Galactose metabolism | 3.516 | 3.819 | < 0.001 |
| map03070 | Bacterial secretion system | -3.356 | -4.259 | < 0.001 |
| map02026 | Biofilm formation - Escherichia coli | 3.335 | 3.515 | 0.001 |
| map01240 | Biosynthesis of cofactors | 3.328 | 3.152 | < 0.001 |
| map00780 | Biotin metabolism | 3.316 | 3.731 | 0.001 |
| map00480 | Glutathione metabolism | 3.229 | 3.584 | 0.001 |
| map01052 | Type I polyketide structures | -3.182 | -3.916 | 0.001 |
| map00290 | Valine, leucine and isoleucine biosynthesis | 2.968 | 3.320 | 0.002 |
| map02010 | ABC transporters | 2.856 | 2.431 | 0.002 |
| map00640 | Propanoate metabolism | 2.836 | 2.954 | 0.002 |
| map00364 | Fluorobenzoate degradation | -2.802 | -3.358 | 0.004 |
| map01212 | Fatty acid metabolism | 2.739 | 3.026 | 0.003 |
| map00540 | Lipopolysaccharide biosynthesis | 2.662 | 2.763 | 0.003 |
| map05230 | Central carbon metabolism in cancer | 2.585 | 2.856 | 0.004 |
| map00061 | Fatty acid biosynthesis | 2.433 | 2.705 | 0.008 |
| map00903 | Limonene degradation | -2.423 | -2.846 | 0.012 |
| map03050 | Proteasome | -2.361 | -2.890 | 0.015 |
| map03050 | Proteasome | -2.361 | -2.890 | 0.015 |
| map01503 | Cationic antimicrobial peptide (CAMP) resistance | 2.237 | 2.387 | 0.012 |
| map00571 | Lipoarabinomannan (LAM) biosynthesis | -2.173 | -2.632 | 0.019 |
| map00040 | Pentose and glucuronate interconversions | 2.121 | 2.127 | 0.014 |
| map00550 | Peptidoglycan biosynthesis | -2.108 | -2.688 | 0.020 |
| map00670 | One carbon pool by folate | 2.079 | 2.225 | 0.016 |
| map00360 | Phenylalanine metabolism | 2.066 | 2.103 | 0.018 |
| map02030 | Bacterial chemotaxis | 2.049 | 2.205 | 0.018 |
| map05017 | Spinocerebellar ataxia | -2.024 | -2.404 | 0.028 |
| map04727 | GABAergic synapse | 2.022 | 2.265 | 0.020 |
| map00630 | Glyoxylate and dicarboxylate metabolism | 2.002 | 2.016 | 0.022 |
| map00907 | Pinene, camphor and geraniol degradation | 1.988 | 2.222 | 0.019 |
| map04151 | PI3K-Akt signaling pathway | -1.981 | -2.405 | 0.029 |
| map05120 | Epithelial cell signaling in Helicobacter pylori infection | -1.978 | -2.338 | 0.028 |
| map00984 | Steroid degradation | -1.935 | -2.409 | 0.029 |
| map00760 | Nicotinate and nicotinamide metabolism | 1.933 | 1.978 | 0.027 |
| map00362 | Benzoate degradation | -1.933 | -2.547 | 0.028 |
| map04122 | Sulfur relay system | 1.907 | 2.092 | 0.025 |
| map00270 | Cysteine and methionine metabolism | -1.837 | -2.457 | 0.037 |
| map00790 | Folate biosynthesis | 1.796 | 1.825 | 0.035 |
| map00510 | N-Glycan biosynthesis | -1.763 | -2.113 | 0.044 |
| map04112 | Cell cycle - Caulobacter | -1.705 | -2.135 | 0.048 |
| map00051 | Fructose and mannose metabolism | 1.657 | 1.551 | 0.048 |
| map00950 | Isoquinoline alkaloid biosynthesis | 1.646 | 1.813 | 0.046 |

**Table S7. Clinical pregnancy outcomes and treatment types among women with PCOS and controls during follow-up.**

| Parameters | PCOS (n=110 ) | Control (n=109 ) | *p* value |
| --- | --- | --- | --- |
| Clinical pregnancy, n (%) | 76/110 (69.1) | 77/109 (70.6) | 0.803 |
| Treatment type, n (%) |  |  |  |
| IVF-ET | 21/76 (27.6) | 24/77 (31.2) | 0.631 |
| IVF-FET | 44/76 (57.9) | 37/77 (48.1) | 0.223 |
| ICSI-ET | 6/76 (7.9) | 8/77 (10.4) | 0.593 |
| ICSI-FET | 5/76 (6.6) | 8/77 (10.4) | 0.564 |
| Biochemical pregnancy, n (%) | 8/76 (10.5) | 2/77 (2.6) | 0.056 |
| Clinical miscarriage, n (%) | 20/76 (26.3) | 8/77 (10.4) | 0.011 |
| Live birth, n (%) | 56/110 (50.9) | 69/109 (63.3) | 0.064 |

Abbreviations: IVF-ET, in vitro fertilisation with embryo transfer; IVF-FET, in vitro fertilisation with frozen embryo transfer; ICSI-ET, intracytoplasmic sperm injection with embryo transfer; ICSI-FET, intracytoplasmic sperm injection with frozen embryo transfer.

Note: One participant in the Control group did not undergo embryo transfer and was therefore excluded from the denominators for treatment type, biochemical pregnancy, and clinical miscarriage analyses.

**Figure S1. Distribution of PCOS phenotypes in the study cohort (n = 110).**

**Figure S2. 16S rRNA sequencing reveals microbiome differences between PCOS patients (n = 30) and Controls (n = 30). (A)** PCoA plots. (**B**) PLS-DA score plot of microbial composition in PCOS and control groups. (**C**) Evaluation of PLS-DA model parameter, Intercept of R2 = 0.819, Intercept of Q2 = -0.283. (**D-G)** Rarefaction curves for the reads number in the PCOS and control groups. Each curve represents one sample and is labeled in pink and blue, respectively. Simpson (D), Chao 1 (E), Shannon (F), and Observed species (G) were used to assess the α-diversity of the gut microbiota. * *p* < 0.05, ** *p* < 0.01.

**Figure S3.** **Correlations between key gut bacterial genera identified by 16S rRNA sequencing and clinical features in all participants.**

**Figure S4. Microbial community differences between PCOS (n = 40) and Control (n = 40) revealed on metagenomic sequencing.** (**A-D**) Indexes of Observed species (**A**), Shannon (**B**), Chao 1 (**C**), and Simpson (**D**) were decreased in PCOS patients. (**E**) PcoA plot. (**F**) Partial Least Squares Discriminant Analysis model applied to metagenomics. Intercept of R2 = 0.804, Intercept of Q2 = -0.234. * *p* < 0.05.

**Figure S5. Association of metagenomic-identified key gut bacterial species with clinical features in all participants.**

**Figure S6. Identification of amino acids and SCFAs as potential molecular markers and their associations with PCOS clinical features.** (**A)** PLS-DA modeling for untargeted metabolomics. Intercept of R2 = 0.740, Intercept of Q2 = -0.290. (**B**) Random forest analysis. (**C**) LASSO analysis identifying key metabolites that best discriminate between groups. (**D**) Correlations between key untargeted metabolites and clinical features in PCOS and Control group. (**E**) The correlation of targeted metabolomic amino acids and SCFAs with clinical features in PCOS and Control group. (**F**) The association of amino acid with SCFA level in PCOS and Control group.

**Figure S7. Receiver operating characteristic curves for serum isoleucine and *P. merdae* abundance in predicting miscarriage.**

**Figure S8. Serum isoleucine levels and *P. merdae* abundance across PCOS phenotypes and Control group.** (**A**) Serum isoleucine concentrations in Control, Phenotype A, and Phenotype D groups. (**B**) Relative fecal abundance of *P. merdae* across the three groups. Different letters indicate statistically significant differences between groups (one-way ANOVA with Tukey post hoc test, *p* < 0.05).

**Figure S9. Transcriptional profile analysis and functional enrichment of differentially expressed genes.** (**A**) Volcano plot of differentially expressed genes. The plot displays the distribution of all genes based on fold change and p-value. Each dot represents a gene. (**B**) Hierarchical clustering heatmap of differentially expressed genes. (**C**) KEGG pathway enrichment analysis of differentially expressed genes.

**Figure S10. Integrative analysis of endometrial senescence in PCOS patients. (A)** Representative H&E staining of endometrial tissues, scare bar = 100μm. (**B)** ESCs were confirmed by vimentin and cytokeratin.

**Figure S11. Correlations between gut microbiota, serum branched-chain amino acids, and SASP factors.** (**A**) Scatter plots showing significant negative correlations between fecal *P. merdae* relative abundance and concentrations of SASP factors IL-1β, IL-6, and IL-8 in ESC-conditioned media. (**B**) Scatter plots demonstrating strong positive correlations between serum Ile levels and SASP factor concentrations. (**C**) Scatter plots showing positive correlations between serum Val levels and SASP factors. (**D**) Scatter plots showing positive correlations between serum Leu levels and SASP factors. Pearson correlation coefficients (R) and *p* values are indicated in each panel.

**Figure S12.** **Effects of *P. merdae* supplementation on adipose tissue and liver in PCOS rats.** (**A**) Representative images of adipose tissue showing quantification of adipocyte area. Scale bar = 100 μm. (**B**) Representative histological images of liver tissue (H&E staining) showing hepatic steatosis in PCOS rats, which was ameliorated by *P. merdae* treatment. Scale bar = 100 μm.

**Figure S13.** **Summary of study findings illustrating the association between gut microbiota dysbiosis, isoleucine accumulation, endometrial senescence, and miscarriage risk in women with PCOS.** Figure S13 was created in BioRender. Jing, M. (2026) https://BioRender.com/undefined.
